# The Short- and Long-Term Risk of Stroke after Herpes Zoster: A Meta-Analysis

**DOI:** 10.1371/journal.pone.0165203

**Published:** 2016-10-21

**Authors:** Xuechun Liu, Yeming Guan, Liang Hou, Haili Huang, Hongjuan Liu, Chuanwen Li, Yingying Zhu, Xingyong Tao, Qingsong Wang

**Affiliations:** 1 Department of Neurology, The 105th Hospital of PLA, Clinic College, Anhui Medical University, Hefei, Anhui Province, People’s Republic of China; 2 Teaching Center of Preventive Medicine, School of Public Health, Anhui Medical University, Hefei, Anhui Province, People’s Republic of China; Fudan University, CHINA

## Abstract

**Background:**

Accumulating evidence indicates that stroke risk may be increased following herpes zoster. The aim of this study is to perform a meta-analysis of current literature to systematically analyze and quantitatively estimate the short and long-term effects of herpes zoster on the risk of stroke.

**Methods:**

Embase, PubMed and Cochrane library databases were searched for relevant studies up to March 2016. Studies were selected for analysis based on certain inclusion and exclusion criteria. Relative risks with 95% confidence interval (CI) were extracted to assess the association between herpes zoster and stroke.

**Results:**

A total of 8 articles were included in our analysis. The present meta-analysis showed that the risks of stroke after herpes zoster were 2.36 (95% CI: 2.17–2.56) for first 2 weeks, 1.56 (95% CI: 1.46–1.66) for first month, 1.17 (95% CI: 1.13–1.22) for first year, and 1.09 (95% CI: 1.02–1.16) for more than 1 year, respectively.

**Conclusion:**

The results of our study demonstrated that herpes zoster was associated with a higher risk of stroke, but the risks decreased along with the time after herpes zoster.

## Introduction

Herpes zoster, a disease evoked by reactivation of latent varicella zoster virus infection, is also known as shingles[1]. Since the population ages, herpes zoster has become a significant public health problem. One million Americans were suffered from it and were frequently complicated by prolonged, severe, disabling pain, and post-herpetic neuralgia every year[2,3].There are other complications of herpes zoster including bacterial skin infection, meningitis, encephalitis, keratitis and herpes zoster ophtalmicus[4,5].

Stroke, bringing huge health and economic burdens, is presently the second leading cause of death globally, especially in countries in Eastern Europe, North Asia, Central Africa, and the South Pacific.[6–8] In China stroke also has outnumbered heart disease to become the leading cause of death and adult disability[9]. Accumulating evidence indicates that following exposure to specific infections, stroke risk is increased. For instance, rates of stroke increased 3-fold after acute respiratory infection[10]. Recent studies have suggested that an episode of herpes zoster may raise risk of stroke[1,5,11–16]. However, the short- and long-term association of herpes zoster and stroke is still not well elucidated. Thus, objective of this study is to systematically analyze and quantitatively estimate connection between the two diseases. Furthermore finding of this meta-analysis may provide reference for future research and help improve clinical practice guidelines.

## Methods

### Search Strategy and Study Selection

Electronic databases (EMBASE, Pub Med, Cochrane library) were searched up to March 2016 using the following terms: herpes zoster or zona or shingles or varicella-zoster virus and cerebrovascular disorders or cerebrovascular accident or stroke or brain ischemia or brain infarction. Searching was limited to human studies and was restricted for all English-language published studies. Additionally, we searched articles from the references within the retrieved articles, and review articles. When there were several retrieved studies from the same study population, only the largest or more recent eligible report was included. Studies were included if they fulfilled the following criteria: (1) studies must be published, peer-reviewed data from an observational study (e.g. a cohort study design); (2) concerned the risks of stroke or transient ischemic attack (TIA) after herpes zoster; (3) reported the multivariate-adjusted relative risks (RRs) and 95% CIs. We pooled data from separate groups when overall estimate was not available. The following excluded criteria were applied: (1) participants had a diagnosis of stroke before the diagnosis of herpes zoster; (2) only reported herpes zoster ophthalmicus; (3) data were insufficient for the calculation of a risk estimate with 95% CI.

### Quality Evaluation and Data Extraction

Quality assessment was based on the Newcastle-Ottawa Scale for our study[17].Two independent reviewers read and scored all of the included studies. The scale consists of 8 items, of which 7 were applicable to our study question. Items were categorized into 3 domains as selection, comparability, and outcome. The total scores of 0–3, 4–5, and 6–8 stars were assigned for low-, moderate-, and high-quality studies, respectively[18]. Any discrepancies between the two reviewers were resolved with a joint reassessment until a consensus was reached.

A predetermined set of data was extracted from eligible study that included the name of the first author, year of publication, design, location, administrative data, age range, following-up time, no of subjects, endpoints, adjusted factors of age and sex, and other adjusted factors. We used the results of the original studies from multivariable models with the most complete adjustment for potential confounders. Based on the reported data in selected studies, we categorized the follow-up time into 4 groups: first 2 weeks, first month, first year, and more than 1 year, respectively.

### Statistical Analysis

Multivariate-adjusted outcome data (expressed as RRs and 95% CIs) were used to estimate the summary statistics. These values were logarithmically transformed in each study and the corresponding standard errors were calculated. The statistical analysis used the inverse variance approach to combine log RRs and standard errors. A random effects model was conducted as it can provide more conservative results than a fixed effects model[19]. A separate analysis using a fixed-effects model was also used and, unless otherwise stated, no differences in the summary estimates were found.

The I^2^ statistic was used to assess heterogeneity. Low, moderate, and high degrees corresponded to I^2^ values of 25%, 50%, and 75%, respectively[20]. The effect of an individual study on the summary estimates was accessed by sensitivity analysis, in which the meta-analysis estimates were calculated omitting one study in turn. Results were presented as pooled RR and their 95% confidence intervals. Moreover, we generated forest plots sorted by level of precision to visually assess RRs across studies. P values were 2 tailed, and the statistical significance was set at .05. The Egger test was used to assess publication bias[21]. All analyses were performed with Stata, version 10.0, software (Stata Corp LP, College Station, Texas).

## Results

### Selected studies and characteristics

**Fig 1:**
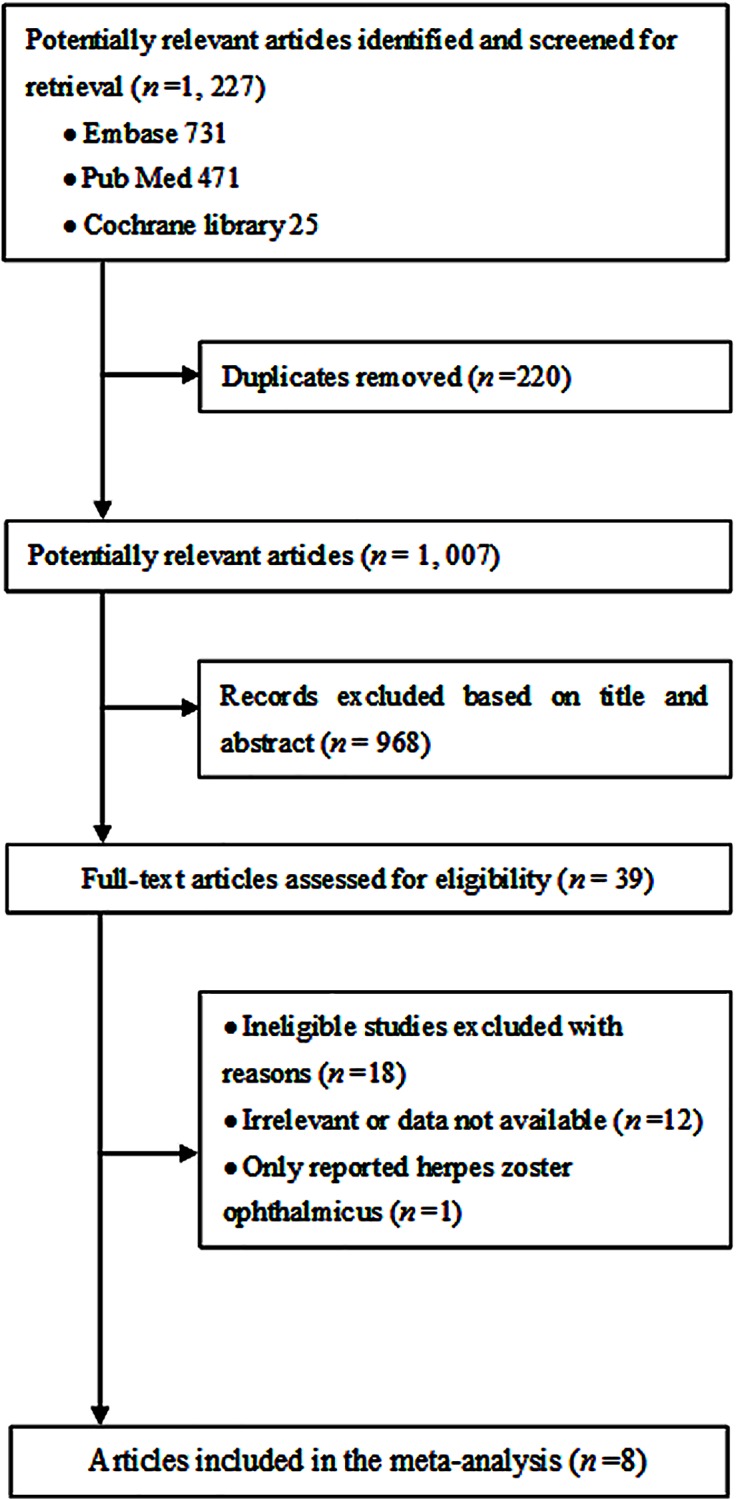
Flow diagram of the search result for the meta-analysis. The figure shows detailed information in the process of search, review, exclusion and inclusion of the potential articles.

Through electronic searches, we identified 1,227 unique articles. After deleting 220 duplicated studies, 968 were excluded on the basis of titles and abstracts, leaving 39 articles for further evaluation. Finally, 8 articles were included in the review after screening full texts (Fig 1) [1,5,11–16].

Of the 8 cohort studies, 2 were from the United States, 1 from Asia and 5 from Europe. The 8 articles were published between 2009 and 2016. The mean follow-up duration in the studies ranged from 1 to 12.5 years, and the sample sizes ranged from 4,862 to 4,620,980 people. All of the included studies were of adequate quality. Two studies received 6 stars, and 6 studies received 7–8 stars, indicating high quality. Adjusted data were available for eight studies. Risk measures concerning age at stroke, and sex were frequently adjusted. The details and characteristics of included studies are listed in Table 1.

**Table 1:** Characteristics of studies included in the meta-analysis.

Author	Year	Design	Location	Administrative data	Age (y)	Following-up time (y)	No of subjects	Endpoints	Controlled factors	ICD	NOS scale
Kwon *et al*[16]	2016	Prospective cohort study	Korea	2003–2013	41.4 (mean)	11	766,179	Stroke, TIA	Age, male gender, hypertension, hyperlipidemia, ischemic heart disease, diabetes, heart failure, peripheral vascular disease, arterial fibrillation or atrial flutter, renal disease, valvular heart disease	ICD-10	7
Yawn *et al*[15]	2016	Community cohort study	United States	1986–2010	68.2 (mean)	7.0 (mean)	4,862	Stroke	Cardiac arrhythmias, a history of vasculopathy, age	ICD-9	8
Minassian *et al*[1]	2015	Self-controlled case series study	United States	2006–2011	≥65	5 (median)	42,954	Ischemic Stroke	Age	ICD-9	7
Sundström *et al*[14]	2015	Population-based cohort study	Sweden	2008–2010	≥0	1	13,296	Stroke	Age, sex	ICD-10	7
Langan *et al*[13]	2014	Self-controlled case series study	United Kingdom	1987–2012	77 (median)	12.5 (median)	6,584	Strok	Age	ICD-10	7
Breuer *et al*[12]	2014	Retrospective cohort study	United Kingdom	2002–2010	57 (median)	6.3 (median)	106,601	Stroke, TIA	Sex, age, obesity, smoking status, history of cholesterol, hypertension,diabetes, ischemic heart disease, atrial fibrillation, intermittent arterial claudication, carotid stenosis, valvular heart disease	ICD-10	8
Sreenivasan *et al*[11]	2013	Population-based cohort study	Denmark	1995–2008	≥18	13	4,620,980	Stroke, TIA	Age, sex, calendar period	ICD-10	6
Kang *et al*[5]	2009	Population-based follow-up study	Taiwan	1997–2001	46.7 (mean)	1	7,760	Stroke, TIA	Age, sex, hypertension, diabetes, coronary heart disease, hyperlipidemia, renal disease, atrial fibrillation, heart failure, heart valve/myocardium disease, carotid/peripheral vascular disease, monthly income, urbanization level, geographical region.	ICD-9	6

TIA, transient ischemic attack; ICD, International Classification of Diseases; NOS, Newcastle-Ottawa Scale; IQR, interquartile range.

**Fig 2:**
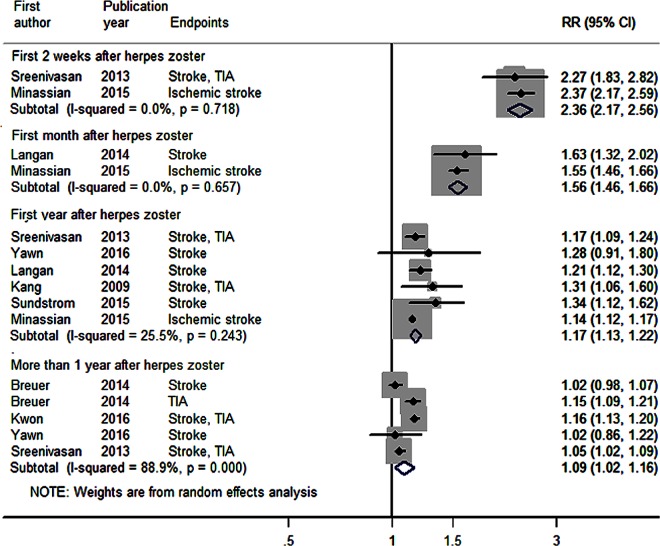
Forest plot of the association between herpes zoster and stroke. The figure shows meta-analysis of the stroke risks after herpes zoster for first 2 weeks, first month, first year, and more than 1 year, respectively. Diamond indicates the overall relative risks (RRs) for the analysis, width of the diamond represents the 95% confidence interval (CI), and boxes represent the weight of individual studies in the pooled analysis.

The results of the meta-analysis are presented in Fig 2. The RR of stroke were 2.36 (95% CI: 2.17–2.56, I2 = 0%) for first 2 weeks, 1.56 (95% CI: 1.46–1.66) for first month, 1.17 (95% CI: 1.13–1.22) for first year, and 1.09 (95% CI: 1.02–1.16) for more than 1 year, respectively. But the I^2^ was 88.9% in the more than one year group, suggesting a high degree of heterogeneity between studies (Q statistic, P<0.001). Sensitivity analyses showed that none of the individual studies dramatically influenced the pooled RR (RR range: 1.07 (95% CI 1.00–1.15) to 1.11(95% CI 1.04–1.18)). No publication biases were detected (P = 0.07 for 0-1year group, P = 0.70 for more than one year group).

## Discussion

To the best of our knowledge, this study is a first meta-analysis examining risk of herpes zoster to the development of stroke. A pooled analysis of 8 observational studies verified that herpes zoster brought a higher risk to stroke; but the risk showed a decreasing trend along with the time after herpes zoster. These results were in accordance with most of related studies. Among studies in the more than one year group, evidence of heterogeneity was detected, which might be partly caused by methodological differences among studies or ethnicities. We abandoned subgroup analysis duo to a few included studies in every groups. More future studies are encouraged on this topic.

The observed increasing risk of stroke following herpes zoster still needs to be explored. Studies show that inflammatory response to virus infection can result in remodeling of vessel wall[22]. And herpes zoster is found able to increase stroke risk through viral invasion of arterial walls and induction of vasculopathy, which leads to thrombosis, occlusions, infarctions, aneurisms and hemorrhages[23]. Further evidence may be attributed to direct infection and viral replication within arterial walls bringing about vascular damage and disruption of vascular flow, which is accompanied by cerebral ischemia or haemorrhage[24]. Besides, inflammation connected with systemic infection may also cause endothelial dysfunction followed by damage of atheromatous plaques and hypercoagulability [25]. As virus replicate in cerebral arteries where infection spread along nerve fibers to blood vessels, thrombotic responses are evoked[26]. Another possible factor is that varicella zoster virus replication adjacent to an artery induces inflammation in the artery and thus increases risk of thrombosis and stroke [27]. In this situation, it is also possible that herpes zoster itself or post-herpetic neuralgia raises sympathetic status and cacoethic emotional reactions, which theoretically increases stroke risk[28]. Research shows that in varicella-zoster virus infected arteries, inflammatory cells secreting soluble factors can potentially disrupt preexisting atherosclerotic plaques [29]. In summary, the modest long term-effect may be explained as a gradual atherosclerotic process evoked by inflammation of vessels due to herpes zoster by infection[5]. More studies are recommended to investigate mechanisms underlying this matter.

This meta-analysis was based on eight studies from various populations. The combined sample size was large and the follow-up period was long in most studies. The estimates from the adjusted models for each study were used in our analyses to reduce the potential of confounders. As a systematic review, our findings and interpretations were limited by the quality and quantity of included studies. Although the potential confounders were considered in most included studies, the residual confounding from unmeasured socioeconomic or other potential factors in prospective cohort studies still cannot be excluded. Due to the changes in International Classification of Diseases codes from the ninth to the tenth revision, the sensitivity and positive predictive value of ICD codes for stroke was more than 82% and 81% in most studies, respectively[30]. The previous study also demonstrated that the simple administrative data studies using a code of herpes zoster can overestimate the 10% to 15% of herpes zoster[31]. So the discrepancies in the definitions of herpes zoster were a potential source of heterogeneity and misclassification. Present TIA being considered as minor strokes, this is a continually controversial topic [32].The potential for publication bias remains due to the searching was limited to all English-language published studies.

The results of current meta-analysis demonstrated that herpes zoster had an adverse effect on stroke, but the risks showed a decreasing trend along with the time after herpes zoster. Further studies are still needed to clarify the association between herpes zoster and stroke in the different age, sex, and ethnicity status.

## Supporting Information

S1 Checklist PRISMA 2009 Checklist. (DOC)
